# Obesity-associated inflammation and cancer: molecular mechanisms, evidence gaps, and therapeutic opportunities

**DOI:** 10.3389/fimmu.2026.1961059

**Published:** 2026-09-21

**Authors:** Jiayi Meng, Xu Bao

**Affiliations:** 1 The Second Affiliated Hospital of Dalian Medical University, Dalian, China; 2 School of Public Health, Dalian Medical University, Dalian, China

**Keywords:** carcinogenesis, inflammation, inflammation signaling pathway, inflammation-targeted therapy, obesity

## Abstract

Obesity, recognized as a significant global public health issue, has garnered widespread attention. The chronic systemic inflammatory state characteristic of obesity may act as a key pathophysiological factor in the development of obesity-related cancers. Based on a structured literature search of peer-reviewed databases, this review summarizes the features of the inflammatory microenvironment within adipose tissue in obesity, encompassing adipocyte hypertrophy and hyperplasia, hypoxia, and endoplasmic reticulum stress; activation of pro-inflammatory cytokines and signaling pathways; and the polarization of immune cells towards a pro-inflammatory phenotype. We emphasize the molecular mechanisms that link inflammation to carcinogenesis and explore targeted therapeutic strategies against inflammation, such as cytokine and receptor blockade, immune cell reprogramming, natural anti-inflammatory agents, and nanoparticle drug delivery systems. This review highlights the mechanistic link between obesity, inflammation, and cancer, providing insights for the development of precise preventive and therapeutic approaches for obesity-associated inflammation and tumorigenesis.

## 1 Introduction

Epidemiological data from the Global Burden of Disease (GBD) 2021 study show that the prevalence of overweight and obesity has increased globally, regionally, and nationally between 1990 and 2021. In 2021, an estimated 1.00 billion adult males and 1.11 billion adult females were living with overweight and obesity (BMI ≥ 25 kg/m²). Without prompt and effective public health interventions, the global burden of overweight and obesity is projected to continue rising, particularly in Asia and Africa, driven by demographic growth, with substantial increases in the number of individuals affected (1). Obesity imposes significant metabolic strain, with research linking it to 21 distinct cardiometabolic, gastrointestinal, respiratory, neurological, musculoskeletal, and infectious diseases (2). Obesity-related insulin resistance is a key pathogenic factor in the development of type 2 diabetes mellitus (2). Concurrently, the risk of multisystem cardiometabolic disorders escalates with increasing BMI (3).

Notably, obesity is closely associated with carcinogenesis. A prospective cohort study involving 3.5 million adults in Spain demonstrated a positive correlation between elevated body mass index (BMI) and nine types of cancers (endometrial carcinoma, renal cell carcinoma, gallbladder carcinoma, thyroid carcinoma, colorectal carcinoma, postmenopausal breast cancer, multiple myeloma, leukemia, and non-Hodgkin lymphoma). Additionally, an association was observed with three other cancers (head and neck cancers, brain and central nervous system tumors, and Hodgkin lymphoma) among never-smokers (4). Another population-based cancer research indicated that 48.47% of 651,342 cancer cases were linked to obesity, with age-standardized incidence rates (ASRs) for 12 obesity-related cancers increasing annually by 3.6% (p < 0.001), while ASRs for non-obesity-related cancers remained stable (5). These findings underscore the importance of addressing obesity as a significant public health concern and emphasize its impact on the global cancer burden.

Obesity is mainly associated with localized adipose tissue and systemic low-grade chronic inflammation, which collectively contribute to the inflammatory microenvironment of adipose tissue and elevated systemic inflammatory cytokines (6). Evidence supports that excess adipose tissue in various anatomical regions promotes inflammation, which can progress to tumor development. In breast tissue, features such as cancer-associated adipocytes, crown-like structures (CLS) surrounding adipocytes, and immune cell infiltration are instrumental in inflammation-driven progression to breast carcinoma (7). In the gastrointestinal tract, increased levels of adipose-derived pro-inflammatory markers such as leptin are linked to abnormal signaling and genetic mutations associated with colorectal cancer (8). Moreover, obesity also contributes to the progression of metabolic dysfunction-associated steatohepatitis (MASH), the inflammatory hepatic subtype of metabolic dysfunction-associated steatotic liver disease (MASLD, formerly non-alcoholic fatty liver disease [NAFLD]), and hepatocellular carcinoma (HCC) through multiple mechanisms (9). In the pancreas, elevated cytokine levels in obese individuals activate ferroptosis and oncogenic signaling pathways, leading to pancreatic ductal adenocarcinoma (10). Furthermore, obesity correlates with heightened inflammatory markers and increased expression of pro-tumorigenic genes, elevating the risk of endometrial carcinoma (11). Overall, chronic inflammation is widely recognized as a contributing factor to carcinogenesis and tumor progression, with mechanisms including dysfunctional repair of inflammation-induced tissue damage, activation of carcinogenic signaling pathways, oxidative stress-induced genomic instability, and immune cell dysfunction (12–15). This review synthesizes recent research on how obesity induces adipose tissue inflammation, elucidating the mechanisms through pro-inflammatory cytokines, inflammatory signaling pathways, immune cell inflammatory states, and unique inflammatory microenvironments, and their links to tumor development. Additionally, we synthesize emerging anti-inflammatory targeted therapies—including novel drugs and mechanisms—which may provide therapeutic benefits for managing cancer in obese patients.

We conducted a structured literature search of PubMed, Web of Science, Embase, and Scopus from database inception to July 2026 to identify peer-reviewed original research articles, systematic reviews, and meta-analyses relevant to obesity-associated inflammation and cancer. The search strategy combined Medical Subject Headings (MeSH) and free-text keywords, including “obesity”, “overweight”, “adipose tissue”, “inflammation”, “pro-inflammatory cytokines”, “adipokines”, “inflammasome”, “tumor microenvironment”, “carcinogenesis”, “cancer risk”, and “anti-inflammatory therapy”, using Boolean operators. The reference lists of the retrieved articles were manually screened to identify additional relevant studies. Articles were included if they were published in English and reported original data or comprehensive syntheses on the molecular mechanisms linking obesity-related inflammation to tumor development, or evaluated inflammation-targeted therapeutic strategies in cancer; conference abstracts, editorials, and non-peer-reviewed sources were excluded. Where clinical evidence was limited, findings from animal models and *in vitro* experiments were included, with explicit acknowledgement of their limitations for extrapolation to humans.

## 2 Characteristics of obesity-associated adipose tissue

Adipose tissue functions as an energy reservoir, and white adipose tissue (WAT) is the most prevalent type of fat storage, with lipids primarily stored as triglycerides (TAG) within lipid droplets (16). Evidence indicates that overnutrition induces a chronic inflammatory response, which may underpin cardiometabolic complications. Mechanistically, the accumulation of adipocytes triggers the activation of metabolic signaling pathways—including c-Jun N-terminal kinase (JNK), nuclear factor kappa-B (NF-κB), Wnt, and Toll-like receptor (TLR)—which further amplify inflammatory processes (17). Additionally, adipose tissue is regarded as an endocrine organ, secreting various hormones and factors collectively known as adipokines. These adipokines play key roles in systemic metabolic regulation, including pro-inflammatory cytokines (e.g., TNF-α, IL-6, IL-1β, IL-8), pro-tumor adipokines (e.g., leptin) and anti-inflammatory cytokines (e.g., adiponectin) (18–21).

Obesity can also trigger adipocyte hypertrophy and hyperplasia, apoptosis, and tissue remodeling, thereby fostering a state of chronic, subclinical inflammation within adipose tissue (22). Enlarged adipose tissue secretes excessive free fatty acids (FFA), reactive oxygen species (ROS), and pro-inflammatory cytokines. The surplus systemic FFAs and dietary lipids infiltrate non-adipose organs such as the liver, muscle, and pancreas, leading to ectopic fat deposition and lipotoxicity. Lipotoxicity can disrupt cellular organelles, such as mitochondria, the endoplasmic reticulum, and lysosomes, leading to the release of excess ROS and pro-inflammatory mediators and, ultimately, to systemic inflammation (23). Moreover, dead adipocytes often become encircled by macrophages, forming crown-like structures that further promote pro-inflammatory phenotypes—a process elaborated upon later. Under severe metabolic stress, hypertrophic adipocytes are prone to undergo pyroptosis, a form of programmed cell death associated with inflammation (24). Adipocyte pyroptosis not only leads to local and systemic inflammation but is also associated with a poor prognosis for cancer treatment (25, 26).

Additionally, endoplasmic reticulum (ER) stress is observed in hepatic steatosis in patients with obesity, serving as a molecular bridge connecting obesity and hepatitis progression (27). ER stress is both a cause and a consequence of inflammatory responses. Despite moderate ER stress helping restore ER homeostasis, persistent or unresolved ER stress in obesity hyperactivates the unfolded protein response (UPR), which ultimately triggers cell death and inflammatory pathways, termed “maladaptive/terminal UPR” (28–30). UPR signaling can directly interfere with downstream inflammatory pathways, including JNK, IKK, and NF-κB signaling cascades, as well as ROS generation (31). This phenomenon manifests as elevated systemic cytokine levels in obese patients, such as TNF-α, IL-6, and IL-1β (32). Interestingly, these inflammatory responses may also function as key mediators of carcinogenesis, causing DNA damage, mutations, and activating pathways such as NF-κB, PI3K/AKT, and MAPK, ultimately enhancing tumor proliferation, migration, and angiogenesis (13, 33).

Another characteristic of adipose tissue in obesity is the rapid expansion-induced hypoxia, where hypoxia-sensitive genes—including GLUT1, MIF, matrix metalloproteinase 9 (MMP-9), PAI-1, and TNF-α—exhibit increased mRNA expression under hypoxic conditions (34). Hypoxia stimulates the expression and secretion of several inflammation-related adipokines, including vascular endothelial growth factor (VEGF), IL-6, leptin, macrophage migration inhibitory factor (MIF), and angiopoietin-like proteins (35). Hypoxia-inducible factor-α (HIF-α) mediates upregulation of these inflammatory adipokines, playing a critical role in hypoxic responses. HIF-α translocates into the nucleus, binds hypoxia response elements within target gene promoters, and activates their transcription—genes involved in angiogenesis, proliferation, survival, apoptosis, vascular tone regulation, and glucose and energy metabolism (36, 37). Interestingly, hypoxia is also a hallmark of the tumor microenvironment (TME), with HIF-α involved in regulating various oncogenic mechanisms. HIF-α promotes the transcription of angiogenic factors such as VEGF, aiding tumor vascularization (14). HIF-α also induces the expression of glucose transporters and glycolytic enzymes, enhancing glycolytic metabolism consistent with the “Warburg effect” observed in cancer cells (38). These observations suggest that the hypoxic microenvironment of adipose tissue may facilitate tumor progression by creating conditions conducive to cancer cell growth in low-oxygen environments.

Importantly, obesity-associated adipose tissue inflammation is not a uniform or monolithic state; its burden varies substantially with metabolic phenotype and fat-depot distribution. In metabolically healthy obesity (MHO), adipose tissue retains a comparatively favorable immune landscape, marked by limited pro-inflammatory macrophage infiltration, a more balanced T-cell repertoire, and reduced levels of inflammatory mediators. In contrast, progression to metabolically unhealthy obesity (MUO) entails progressive immune-cell remodeling, adipokine dysregulation (rising leptin with declining adiponectin), and escalating systemic low-grade inflammation (39, 40). Building on this phenotypic heterogeneity, individual adipose depots display distinct inflammatory biology: visceral adipose tissue (VAT), characterized by high immune-cell density, high lipolytic flux, and direct portal drainage, serves as a dominant source of systemic inflammation and metabolic risk; subcutaneous adipose tissue (SAT) is comparatively metabolically protective, although its buffering capacity erodes under prolonged nutrient excess; and brown adipose tissue (BAT), while thermogenically and metabolically beneficial, undergoes whitening and functional decline in obesity, further amplifying low-grade inflammation (41–44). These observations argue against treating adipose inflammation as a binary process and underscore that metabolic phenotype and fat distribution, rather than body mass index alone, should be integrated when interpreting inflammatory signatures and their consequences.

## 3 Pro-inflammatory components in obesity

### 3.1 Pro-inflammatory cytokines and inflammatory signaling pathways

In WAT, apart from adipocytes, which are the most abundant cell type, the tissue also comprises endothelial cells, fibroblasts, leukocytes, and notably, macrophages. Adipose tissue macrophages are a major source of TNF-α in obesity, whereas the adipose tissue—mainly via its infiltrating immune cells rather than mature adipocytes—contributes an estimated 15–35% of circulating IL-6, reaching approximately one-third in obese individuals (45, 46). Both cytokines can activate numerous pro-inflammatory signaling pathways, thereby contributing to the inflammatory state of the adipose tissue (47). TNF-α activates downstream signaling pathways such as mitogen-activated protein kinase (MAPK) and NF-κB, thereby directly promoting inflammatory processes. This activation leads to increased expression of a variety of chemokines and inflammatory mediators (48). Furthermore, TNF-α is involved in the activation of additional signaling cascades, including JNK, IKK, and JAK pathways, ultimately integrating into immune-metabolic programs (49). NF-κB is a crucial inflammatory signaling pathway, serving as the common downstream effector for multiple inflammatory mediators including TNF-α and IL-1 (50). In canonical signaling pathways, NF-κB/Rel dimers are maintained in an inactive state within the cytoplasm through association with IκB inhibitors or interactions with unprocessed NF-κB1 and NF-κB2 precursors. Obesity-induced pro-inflammatory cytokines can activate the IKK complex, comprising IKKβ, IKKα, and IKKγ (NEMO), leading to phosphorylation and subsequent proteasomal degradation of IκB proteins, and the release of NF-κB for nuclear translocation (51, 52). Activated NF-κB undergoes further phosphorylation and translocation to the nucleus, where it induces the transcription of target genes involved in inflammatory responses, including pro-inflammatory cytokines, chemokines, and growth factors including TNF-α, IL-1, IL-6, and vascular endothelial growth factor, thereby sustaining chronic inflammation (53). Upon binding to cytokines (e.g., IL-6), JAKs undergo phosphorylation on specific tyrosine residues, thereby activating signal transduction and activators of transcription, known as STATs. The activation and dimerization of STAT proteins reveal their nuclear localization signals, allowing them to translocate to the nucleus. Once there, they bind to specific enhancer elements within the promoters and enhancers of target genes, initiating the transcription of inflammatory genes (54, 55). Additionally, IL-6-induced activation of PI3K modulates the mTOR complex 1 (mTORC1) pathway, impacting various cellular processes such as metabolism and redox homeostasis (56).

### 3.2 Pro-inflammatory molecules

Interferon (IFN) family members, particularly IFN-γ, play a crucial role in obesity, inflammation, and immune regulation. Elevated expression of IFN-γ has been observed in adipose tissue correlating with diet-induced obesity (DIO) murine models (57). Additionally, experimental data demonstrate that IFN-γ deficiency results in a significant reduction in the expression of inflammatory cytokines and chemokines, such as monocyte chemoattractant protein-1 (MCP-1) and IL-10, indicating that IFN signaling modulates the inflammatory gene expression within adipose tissue (58). The increased levels of IFN-γ activate immune cells, promoting their inflammatory phenotype and recruiting various immune cell subsets—including T lymphocytes, macrophages, and natural killer (NK) cells—to adipose tissue (58–60). This immune activation contributes to local and systemic inflammatory responses, which can facilitate the development of obesity-related comorbidities such as insulin resistance and MASLD (61, 62).

Leptin, a 16-kDa cytokine primarily secreted by white adipocytes, exerts anorexigenic effects and augments energy expenditure. It is also recognized as a potent pro-inflammatory, proliferative, and anti-apoptotic mediator, serving as a key regulator in nutrient sensing, metabolic processes, and immune homeostasis (63). Substantial evidence indicates that obese individuals exhibit elevated circulating leptin levels (64). The pro-inflammatory effects have been demonstrated to promote the production of cytokines, such as TNF-α, IL-6, and IL-12, through the JAK-STAT, MAPK, and PI3K pathways. Exposure to inflammatory stimuli like TNF-α and IL-1 increases leptin levels and expression in adipose tissue, establishing a positive feedback loop that further amplifies inflammation through mutual interactions with other cytokines (47, 65). In addition, leptin can affect specific immune responses that further exacerbate inflammation. Leptin-mediated activation of M1-like macrophages significantly enhances CXCL2 production and neutrophil recruitment by modulating the JNK/STAT3/AKT signaling pathways, concurrently elevating levels of Th1/Th17-associated inflammatory cytokines and promoting pro-inflammatory M1 macrophage activation states (66). Despite its essential role in early T cell development and the maturation of CD4^+^ T cells, leptin significantly contributes to the promotion of immune cell-driven inflammatory responses, playing a crucial role in the pathogenesis of obesity-related diseases (67). Moreover, chronic hyperleptinemia is accompanied by leptin resistance, which hinders the therapeutic efficacy of obesity-related metabolic disorders (68). However, leptin should not be viewed as a unidirectional pro-inflammatory mediator, as the net impact of leptin signaling on inflammation and tumor immunity varies across tissues, disease stages, and tumor types (69, 70).

Interestingly, these adipose-derived inflammation-associated signaling molecules and pathways also drive tumorigenesis, given that tumor cells express leptin and diverse cytokine receptors. When these receptors bind to their respective molecules, the activation of NF-κB, MAPK, JAK-STAT, and PI3K-AKT pathways is triggered, thereby promoting the proliferation, migration, and invasion of malignant cells (71, 72). In cases of acute inflammation, NF-κB acts as an ally in the clearance of pathogens and tissue repair. However, in chronic conditions, it frequently assumes the role of a “villain” by maintaining sustained activation. This can lead to the initiation of anti-apoptotic gene expression, promotion of cellular proliferation, stimulation of angiogenesis, induction of epithelial-to-mesenchymal transition, and facilitation of cancer cell metastasis and invasion (73–75). In summary, given the shared signaling pathways between obesity-associated inflammation and tumorigenesis, targeting these pro-inflammatory mediators may represent a promising strategy for developing therapeutic agents against cancers in obese patients.

### 3.3 Inflammasome

Inflammasomes are multiprotein complex assemblies that serve as innate immune sensors and are considered key mediators of inflammatory responses. Pathogen-associated molecular patterns (PAMPs), such as lipopolysaccharide (LPS), activate pattern recognition receptors (PRRs) like TLR4, initiating the NF-κB signaling pathway and inducing transcription of NLRP3 and IL1B (encoding pro-IL-1β) (76). Subsequently, various damage-associated molecular patterns (DAMPs) act as secondary signals, promoting NLRP3 oligomerization and ASC speck formation (77). Activated NLRP3 then recruits the adaptor protein ASC via its pyrin domain (PYD), which in turn, through CARD-CARD interactions, recruits multiple pro-caspase-1 molecules to assemble a functional NLRP3 inflammasome complex (78). Activated caspase-1 not only processes pro-IL-1β and pro-IL-18 into their mature, secreted forms but also cleaves Gasdermin D. The N-terminal fragment of Gasdermin D oligomerizes on the cell membrane to form pores, initiating pyroptosis, a form of programmed necrotic cell death characterized by cell swelling, membrane rupture, and the release of inflammatory mediators, thereby amplifying the local inflammatory response (79).

In addition to NLRP3, which senses a broad range of metabolic and sterile danger signals predominantly in macrophages and, to a lesser extent, adipocytes, other canonical inflammasomes are activated by distinct ligands and show distinct cellular distributions (80). The NAIP–NLRC4 inflammasome responds to cytosolic flagellin and type III secretion system (T3SS) rod/needle proteins in macrophages and intestinal epithelial cells, whereas AIM2 recognizes cytosolic double-stranded DNA of microbial or host origin and is expressed in macrophages, dendritic cells, keratinocytes, and intestinal epithelial cells (81, 82). Upon assembly, both NLRC4 and AIM2 recruit ASC and caspase-1 and elicit IL-1β/IL-18 maturation and gasdermin D-dependent pyroptosis, mirroring the downstream machinery of NLRP3 (83). Notably, NLRC4 and AIM2 also exert inflammasome-independent functions—including NLRC4-mediated DNA damage responses and AIM2-dependent transcriptional regulation—and their roles in obesity-associated inflammation and tumor immunity are increasingly recognized (84, 85).

Inflammasome activation has been implicated as an important contributor to obesity-associated inflammation, particularly in preclinical models. Inflammasomes sense cellular stress elicited by lipotoxic intermediates, including ceramides, within adipocytes, triggering NLRP3 activation through mechanisms involving autophagy impairment and ROS accumulation (86, 87). This activation subsequently promotes Th1 pro-inflammatory activation states and M1-like macrophage activation, contributing to inflammatory processes (88). Additionally, ER stress induced by obesity can activate inflammatory responses via the NLRP3 inflammasome pathway (89). Further research confirms that a high-fat diet (HFD) induces NLRP3 inflammasome activation, immune cell infiltration in glomeruli, and increased release of pro-inflammatory extracellular vesicles. Circulating TNF-α levels in obese individuals can also induce, in an NLRP3-dependent manner, the expression and secretion of lipocalin-2 (Lcn2), further amplifying macrophage-driven inflammation (90, 91). Evidence of inflammation mediated by inflammasomes in obesity also includes findings that inhibitors of the NLRP3 inflammasome downregulate CCL2, IL-1β, and IL-6, thereby reversing HFD-induced obesity and systemic inflammation in rodent models (92, 93). **Figure 1** illustrates the major inflammatory signaling pathways associated with adipose tissue in obesity.

**Figure 1:**
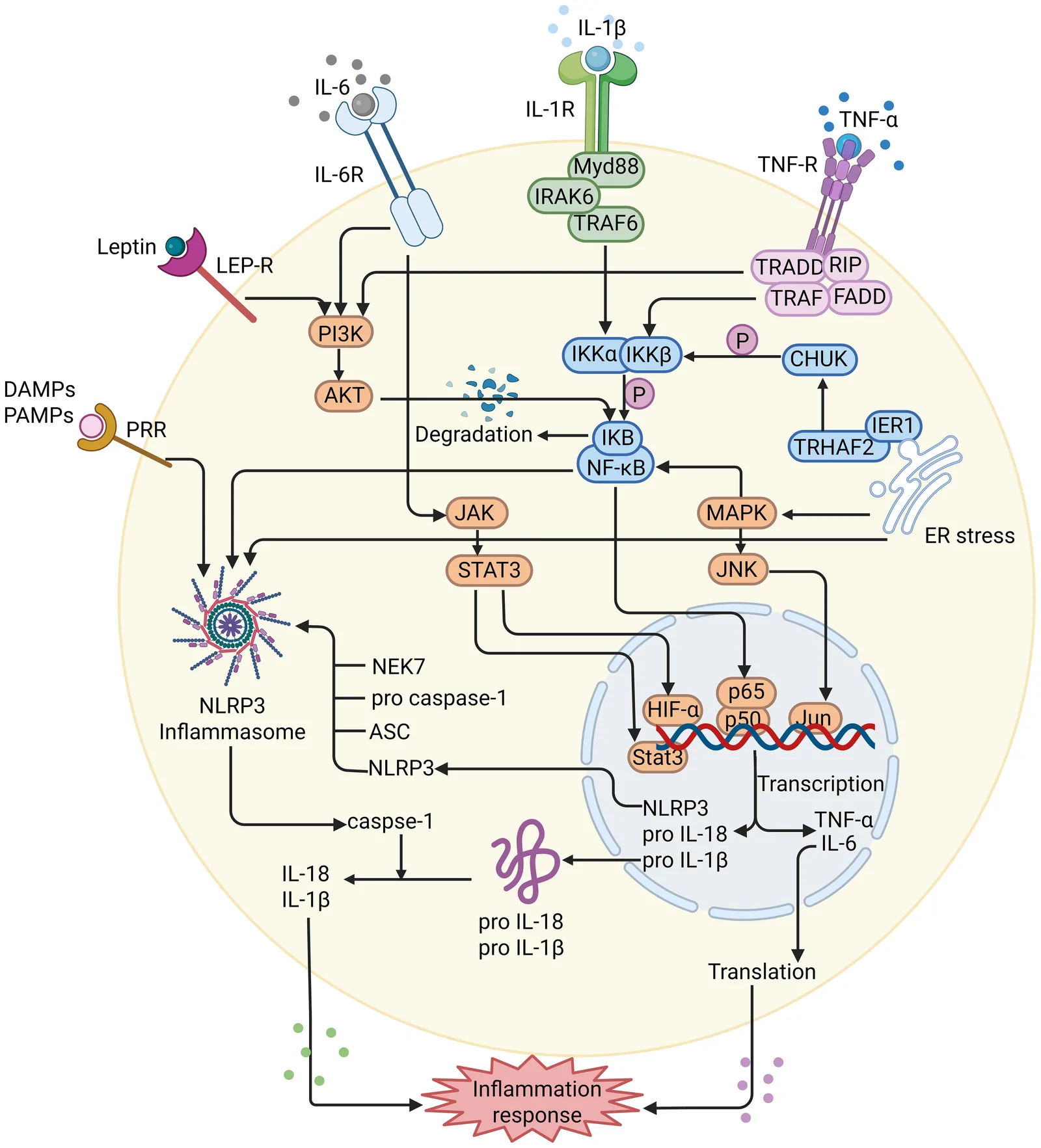
Inflammatory signaling pathways in obese patients. Elevated levels of circulating inflammatory cytokines (e.g., IL-6, IL-1β, TNF-α), along with increased leptin and responses to DAMPs triggered by tissue metabolic imbalance, may activate relevant inflammatory signaling pathways in obese patients. Key molecular mechanisms underlying the inflammatory response are depicted, including NF-κB, PI3K/AKT, and JAK/STAT3. These pathways activate nuclear transcription of inflammatory cytokine genes (e.g., IL-6 and TNF-α), further exacerbating inflammation in obese patients. Additionally, the assembly and activation of the NLRP3 inflammasome are represented, which promotes the production and release of inflammatory cytokines IL-1β and IL-18. Besides, ER stress also contributes to the inflammatory signaling and inflammasome pathway. The pathways described above clearly illustrate how inflammation leads to further inflammation in obese individuals, resulting in persistently elevated levels of inflammation within the body. DAMPs, damage-associated molecular patterns; PAMPs, pathogen-associated molecular patterns; PRRs, pattern recognition receptors; ER, endoplasmic reticulum; MAPK, mitogen-activated protein kinase.

Accumulating evidence has established a critical link between NLRP3 inflammasome activation and tumorigenesis. Pan-cancer analyses indicate that, among 24 cancer types, 15 exhibit significant alterations in the expression of NLRP3 inflammasome-related genes, suggesting that NLRP3 inflammasomes may primarily modulate tumor immunity by mediating infiltration of lymphocytes and macrophages (94). Some hypotheses propose that inflammasome-induced secretion of cytokines IL-1β and IL-18 may favor an immunosuppressive TME in certain settings (e.g., by recruiting MDSCs), whereas their net impact on NK- and T-cell-mediated immune surveillance is context-dependent (95). Importantly, in the context of obesity, the TME may induce increased infiltration of myeloid cells through activated NLRC4 inflammasomes. This subsequently activates IL-1β and drives breast cancer progression by promoting adipocyte-mediated vascular expression of VEGFA and angiogenesis (96). Conversely, other perspectives suggest that NLRP3 plays a protective role in certain tumors, maintaining intestinal homeostasis and preventing colorectal carcinogenesis through its critical functions in suppressing colitis and maintaining gut immune balance (97). However, NLRP3 activation can either promote or restrain tumor progression depending on tumor type, stage, and microenvironmental context. For example, colitis-associated colorectal cancer can be suppressed by NLRP3-dependent IL-18 production, whereas gastric and skin cancers may be promoted by NLRP3-driven inflammation (45). In gynecological malignancies, NLRP3 contributes to immunosuppression in certain tumor types or molecular subtypes while enhancing pyroptosis-associated chemosensitivity in others (98). Collectively, these context-dependent dual functions render the NLRP3 inflammasome a double-edged regulator in tumorigenesis.

Several inflammasome-targeted therapeutics—including inhibitors of NLRP3, caspase-1, and IL-1β—have been developed and are being evaluated for cancer treatment (99). However, challenges persist regarding adverse effects, drug resistance, and individual variation across cancer types, underscoring the need for further research to determine whether obesity influences the efficacy of inflammasome inhibitors. Moreover, clinical evidence for inflammasome-targeted therapy in obese patients is still limited, with most evidence originating from rodent experiments. Given interspecies differences in inflammasome function and immune-metabolic control, these preclinical results are not readily transferable to humans. Nevertheless, animal data offer valuable mechanistic insights, illuminate key molecular cascades connecting obesity-related inflammation to cancer, and guide the selection of targets for subsequent clinical investigation. **Table 1** summarizes key inflammatory cytokines, signaling pathways, and mediators in the adipose tissue of obese patients.

**Table 1:** Key inflammatory cytokines, signaling pathways, and mediators in the adipose tissue of obese patients.

Category	Component/pathway	Role in obese adipose tissue	Mechanistic link to carcinogenesis	References
Pro-inflammatory Cytokines	TNF-α	Hyperactivated in adipocytes; drives ER stress and tissue inflammation	Activates MAPK and NF-κB signaling; promotes tumor proliferation, survival, and tissue remodeling	(32, 47, 48)
	IL-6	Secreted abundantly by adipocytes and infiltrating immune cells	Activates JAK/STAT3; modulates PI3K/mTORC1 metabolism; fuels tumor growth and anti-apoptosis	(32, 47, 56)
	IL-1β	Cleaved and matured via inflammasome under metabolic stress	Sustains pro-inflammatory microenvironment; impairs cytotoxic T-cell surveillance; recruits immunosuppressive cells	(32, 79, 90, 92)
Inflammatory Signaling Pathways	NF-κB Pathway	Canonical cascade activated by TNF-α, IL-1, and TLR4 stimulation	Transcribes anti-apoptotic genes; induces EMT, angiogenesis, and metastasis	(17, 31, 50, 51, 73, 74)
	JAK/STAT3	Phosphorylated and activated upon IL-6 or leptin receptor binding	Nuclear translocation of oncogenic genes; facilitates tumor invasion and immune evasion	(54, 55, 66, 72)
	PI3K/AKT/mTORC1	Stimulated by nutrient surplus, saturated fatty acids, and adipokines	Integrates metabolic signals to enhance glycolytic flux and cell growth; biases CD4^+^ memory T-cell differentiation	(33, 56, 72)
Bioactive Mediators & Receptors	Leptin/obR axis	Secreted by hypertrophic adipocytes; systemic appetite regulator with pro-inflammatory activity	Promotes M1 macrophage polarization and CXCL2 release; directly drives tumor PI3K/AKT and MAPK pathways	(20, 63, 64, 66, 71)
	IFN-γ	Highly expressed in diet-induced obesity; recruits macrophages, T lymphocytes, and NK cells	Drives chronic tissue damage and MASLD progression; induces PD-L1 expression to exhaust CD8^+^ cytotoxic T cells	(57, 58, 62)
Cellular Stress & Death Complexes	NLRP3 Inflammasome	Triggered by lipotoxic ceramides, ER stress, and DAMPs	Promotes maturation of IL-1β and IL-18; induces pyroptosis; recruits myeloid-derived suppressor cells (MDSCs)	(76, 79, 87, 89, 95, 96)
	HIF-1α/Hypoxia-sensitive genes	Rapid fat expansion creates hypoxia; upregulates GLUT1, MIF, VEGF, and PAI-1	Mimics hypoxic tumor microenvironment; induces angiogenesis and metabolic reprogramming toward the Warburg effect	(14, 34–36, 38)

## 4 Inflammatory response of immune cells

### 4.1 Inflammatory conditions of immune cells

#### 4.1.1 Macrophages

Obesity has been proven to not only elevate levels of pro-inflammatory cytokines but also to promote inflammatory alterations in immune cells, both of which contribute to inflammatory diseases. Recent scientific advancements have provided new insights into the relationship between obesity and immune function, revealing that excess adiposity induces metabolic remodeling in WAT, leading to immune cell infiltration and, consequently, systemic secretion of pro-inflammatory mediators. Therefore, adipose tissue can be regarded as an immunometabolic and endocrine organ, while obesity is characterized as an inflammatory immunopathology (100).

Macrophages are essential components of the innate immune system and play a critical role in inflammatory processes by secreting cytokines such as TNF-α, IL-6, IL-8, and CCL2. Macrophages exhibit high plasticity, enabling rapid phenotypic alterations through activation states in response to local microenvironment stimuli (101). Based on stimuli, surface molecule expression, cytokine secretion profiles, and functional characteristics, macrophages are commonly described as classically activated M1-like or alternatively activated M2-like (102). Macrophages also exist in a non-polarized, M0 state, which, upon activation by various cytokines, differentiates into M1- or M2-like phenotypes (103). M1-like macrophages are generally pro-inflammatory, induced by Th1 cytokines such as IFN-γ and TLR ligands like LPS. They are characterized by the production of pro-inflammatory cytokines (e.g., IL-6, IL-1β, TNF-α) and inducible nitric oxide synthase (iNOS) expression (104). Conversely, M2-like macrophages are primarily activated by IL-4 and IL-13, favoring anti-inflammatory responses, secreting IL-10 and arginase-1 (Arg-1), and upregulating chemokines such as CCL17 and CCL24, contributing to tissue repair through the release of anti-inflammatory cytokines (e.g., IL-10, IL-12, TGF-β) (104, 105).

Analysis of macrophages in adipose tissue of obese mice reveals a predominantly M1-like phenotype, with the M2 to M1 ratio shifting from 4:1 in lean animals to approximately 1.2:1 in obese subjects (106). Adipose tissue macrophage activation states are further influenced by lipid and glucose accumulation, which are markedly elevated in obesity. These metabolites promote M1-like activation states through mechanisms involving TLR4, Rho-associated kinase (ROCK)/JNK pathways, and adipocyte-derived microvesicles (ADM), thereby reinforcing the inflammatory milieu (107). This activation pattern corresponds with obesity-related inflammation, which is driven by the secretion of pro-inflammatory cytokines, thereby exacerbating systemic inflammatory responses.

It should be emphasized, however, that this binary classification is largely derived from in vitro stimulation and oversimplifies the continuous, tissue- and context-dependent activation states of macrophages observed in vivo (108). Single-cell transcriptomic and multi-omics studies have revealed that ATMs do not exist as fixed M1 or M2 states in vivo, but instead comprise multiple subsets with distinct origins (tissue-resident vs. bone marrow-derived monocyte-recruited), spatial localization, metabolic activation states, and functions (109, 110). Recent studies suggest that, under lipid-rich, glucose-rich, and insulin-rich adipose tissue conditions, the macrophage activation states are better described by the metabolically activated macrophage (MMe) phenotype rather than the traditional M1/M2 classification. MMe macrophages exhibit distinct surface markers different from classical M1 or M2 phenotypes, with their marker expression reflecting activation pathways specific to the local microenvironment, rather than serving as definitive identifiers of cell phenotypes (111, 112). Emerging subtypes of adipose tissue macrophages, such as CD9-positive and Trem2-high macrophages, indicate that lipid excess triggers responses in macrophages that compromise tissue homeostasis, potentially advancing inflammation and metabolic disease progression (112, 113). Moreover, a mitochondrial-stress circuit between AD3 adipocytes and the LAM-ST1 macrophage subset sustains metaflammation in human type 2 diabetic adipose tissue (114). Overall, macrophage activation states are a complex, environment-dependent process, characterized by distinct activation pathways and functional states depending on contextual stimuli. While macrophages can exert pro-inflammatory effects during inflammatory progression, their activation states are dynamically adaptable to maintain organismal homeostasis.

#### 4.1.2 T cells

Furthermore, immune cells such as T lymphocytes, dendritic cells, and neutrophils can actively respond to adipose tissue inflammation, differentiating into pro-inflammatory phenotypes under obesity conditions. In obese adipose tissue, pro-inflammatory immune cells—including CD8+ T cells, Th1 cells, M1-like macrophages, neutrophils, and mast cells—accumulate, whereas cells generally associated with anti-inflammatory or tissue-repair functions—such as regulatory T cells (Tregs), Th2 cells, and M2-like macrophages, together with eosinophils and group 2 innate lymphoid cells (ILC2s)—decrease (112). Notably, in HFD models, CD4^+^ T cells tend to adopt a hyperactivated, pro-inflammatory phenotype characterized by increased PI3K p110δ-Akt signaling. The specific phenotype is CD44 hi-CCR7 lo-CD62L lo-CXCR3+–LFA1+ (115). Extracellular assays revealed an increased presence of pro-inflammatory Th1 and Th17 cells in adipose tissue, accompanied by a decrease in anti-inflammatory Tregs, leading to an imbalance between the Treg and Th17 populations (116, 117). In the context of obesity, Th1 cells may exert inhibitory effects on Th2 cells through the production of IFNγ. Other molecules, including leptin and adiponectin, may also contribute to the suppression of Th2 cell activity during obesity, thereby promoting a pro-inflammatory Th1 skewing (117). Notably, the metabolically stressed adipose microenvironment in obese patients (e.g., saturated fatty acid overload, diminished IL-33/ST2 signaling, and upregulated OX40L) dynamically reshapes the abundance and function of Th2 and Treg cells, converting them from homeostatic regulators into functionally impaired or even pro-inflammatory populations, underscoring the highly environment-dependent nature of these cells (118–121).

Moreover, macrophages in HFD states produce chemokines like CCL5 and CXCL10, along with pro-inflammatory cytokines including TNF-α, IL-1β, IFN-γ, and IL-17A, which further activate T cells. Conversely, T cells from HFD-fed mice can induce the expression of inflammatory genes in ND macrophages and increase lipid accumulation in 3T3-L1 adipocytes, along with the heightened production of inflammatory cytokines such as CXCL2, CCL3, and CCL4 (117). This highlights the collaborative role of macrophages and T cells in maintaining chronic inflammation within the body.

#### 4.1.3 Dendritic cells

Studies of dendritic cells (DCs) indicate that HFD induces plasmacytoid dendritic cell (pDC) activation within visceral adipose tissue (VAT), resulting in elevated type I interferon (IFN-I) expression. Conversely, IFN-I facilitates the shift of M2-like macrophages toward M1-like activation states (122). DCs may mediate tissue inflammation through the expression of molecules such as MIF, IL-18, IL-1β, IL-12, CCL5, MCP-3, and TNF-α (122, 123). In the context of obesity, tissue-resident natural killer (trNK) cells participate in cDC1 recruitment via XCL1/2-XCR1 signaling, similar to mechanisms recently documented within the TME. Additionally, cDC2B subsets potentially promote tissue inflammation through the expression of MIF, IL-18, IL-1β, and TNFSF9 (124).

#### 4.1.4 Neutrophils

Compared to lean subjects, neutrophils within VAT are more abundant in obese individuals and exhibit higher expression levels of inflammation- and activation-related genes than peripheral blood (PB) neutrophils. The increase in neutrophils is followed by a rise in pro-inflammatory CD4^+^ Th1 cells and a decrease in anti-inflammatory Tregs (125). Obesity-associated inflammation and oxidative stress may also facilitate neutrophil degranulation and the release of chromatin structures, similar to the formation of neutrophil extracellular traps (NETs), which trap pathogens but may also promote further neutrophil recruitment, perpetuating a vicious cycle of chronic inflammation (126).

This sustained inflammatory response ultimately contributes to tumor progression. For T lymphocytes, chronic inflammation suppresses immune functionality through the programmed cell death protein 1 (PD-1) and its ligand PD-L1 pathway, resulting in exhaustion of CD8+ and CD4^+^ T cells. Persistent inflammatory stimuli induce macrophage activation states toward immunosuppressive, M2-like activation states (127). In addition, inflammatory signals can reprogram fibroblasts into cancer-associated fibroblasts (CAFs), which facilitate angiogenesis and extracellular matrix (ECM) remodeling—processes that promote tumor invasion and metastasis (128). Furthermore, infiltration of immune cells, including tumor-associated macrophages, immature myeloid cells, and T cells, contributes to a pro-tumorigenic inflammatory microenvironment, exacerbating the vicious cycle of adipose tissue inflammation, immune cell recruitment, and tumor promotion (129). **Figure 2** illustrates the crosstalk between the inflammatory microenvironment of adipose tissue and the TME via molecules and inflammatory immune cells. It is generally accepted that obesity activates and polarizes various immune cells toward a pro-inflammatory phenotype; these polarized cells then release cytokines that amplify systemic inflammation in obese patients. This chronic inflammatory state and its associated risks may represent a potential mechanism underlying tumorigenesis. The inflammatory microenvironment within obese adipose tissue can crosstalk with the TME through inflammatory immune cells and cytokines, representing a possible pathway linking obesity to cancer development. Therefore, strategies aimed at preventing pro-inflammatory activation states of immune cells and modulating immune cell subtypes to prevent biased pro-inflammatory responses may offer therapeutic benefits in reducing systemic inflammation and improving cancer outcomes in obese individuals.

**Figure 2:**
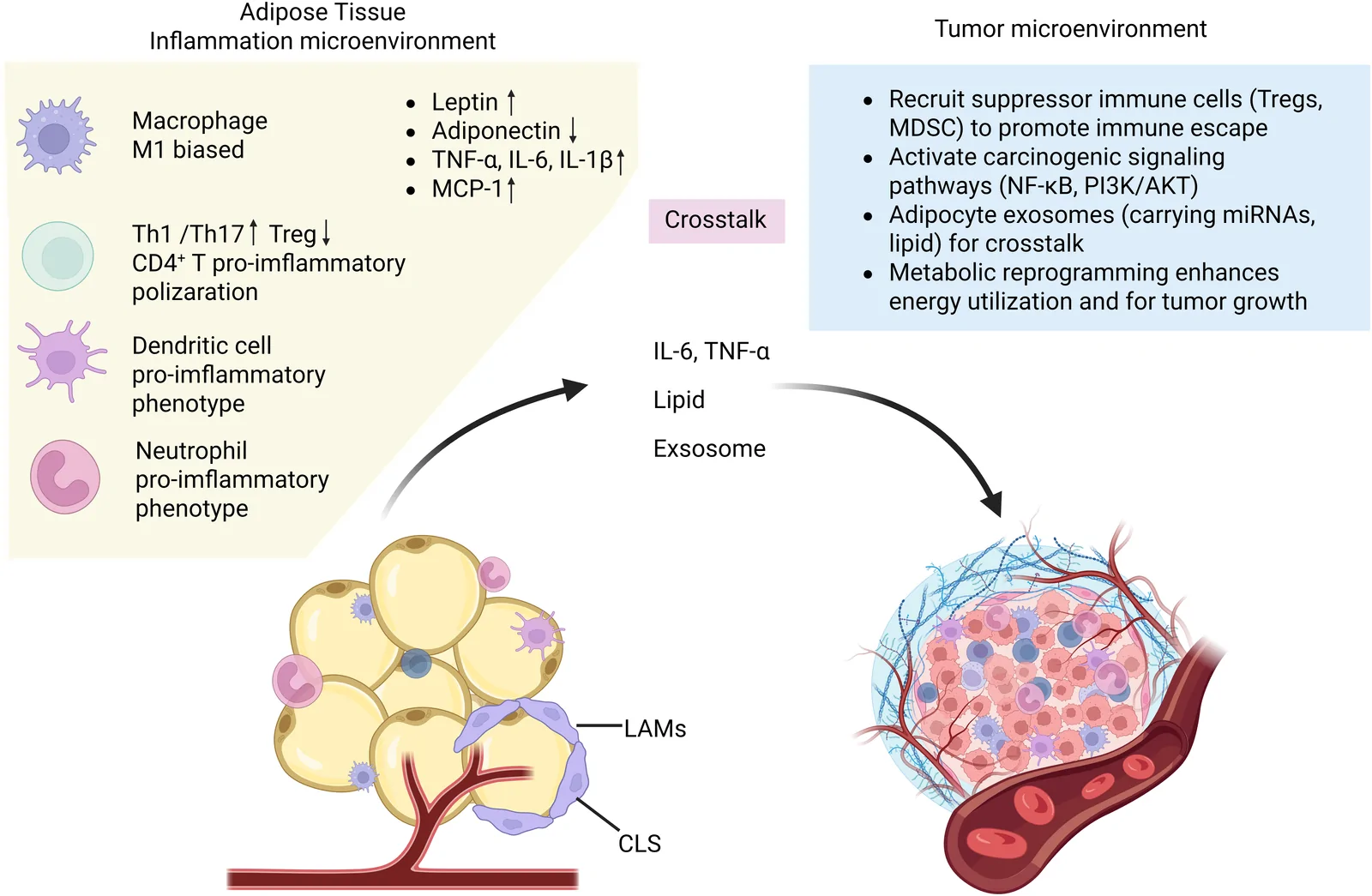
Crosstalk between adipose tissue inflammation microenvironment and TME. The adipose tissue inflammation microenvironment is shown, containing immune cells such as M1-like macrophages, CD4^+^ T cells with context-dependent inflammatory phenotypes (including Th1, Th2, and Treg subsets), dendritic cells, and neutrophils. Besides, there are also imbalanced hormone levels (elevated leptin and reduced adiponectin), and increased levels of cytokines and chemotactic factors. Additionally, the figure illustrates the CLS formed by macrophages surrounding necrotic adipocytes, including lipid-associated macrophages. The crosstalk between the inflammation microenvironment and the TME occurs via IL-6, TNF-α, lipids, and exosomes. In the TME, some carcinogenesis mechanisms are depicted as the downstream events after the crosstalk, and then promote tumor growth and metastasis. CLS, crown-like structure; LAM, lipid-associated macrophage; MCP-1, monocyte chemoattractant protein 1; TME, tumor microenvironment.

### 4.2 Crown-like structures in adipose tissue

A substantial influx of mononuclear cells has been observed in the adipose tissue surrounding necrotic adipocytes, which differentiate into pro-inflammatory M1-like macrophages. These macrophages form “crown-like structures” (CLS) around individual adipocytes, serving as histological biomarkers of inflammation. Some researchers consider adipocyte death to trigger a unique degradation process that locally induces M1-like polarization-associated genes, thereby serving as the origin of chronic inflammation in obesity (130, 131).

The formation of these distinctive histological CLS relies on two primary factors: adipocyte necrosis and macrophage recruitment. Adipocyte necrosis may result from metabolic dysregulation within adipose tissue, where excessive lipid accumulation leads to hypertrophy and hyperplasia, forcing cell death due to overload (132). It may also stem from obesity-induced tissue inflammation, mediating adipocyte necroptosis (133). Additionally, studies have observed the release of DAMPs and chemokines within CLS environments, including MCP-1, prostaglandins, and leukotrienes, which promote activation, proliferation, and recruitment of pro-inflammatory phagocytic macrophages into adipose tissue (134). In DIO models, the reduced expression of PPARγ in Tregs exacerbates the infiltration of pro-inflammatory macrophages and monocytes, thereby fostering an environment that facilitates the formation of CLS (135).

The beneficial role of CLS includes participation in lipid clearance (136). However, CLS primarily serve as indicators of adverse effects, with adipocytes within CLS expressing pro-inflammatory cytokines (e.g., TNF-α and IL-6), implying a pro-inflammatory lesion (137). In addition, CLS are associated with the development of certain cancers, notably breast cancer. Specifically, the abundant fat pads surrounding the lymph nodes in the breast, along with their close association with adipose tissue and macrophages, suggest a connection (138). A study utilizing existing cohorts of early-stage breast cancer patients demonstrated that the presence of CD68+ CLS-B signifies a phenotype connecting obesity, macrophage-mediated inflammation, and breast cancer (139). Moreover, early evidence indicates that CLS-B contributes to breast carcinogenesis and progression through mechanisms involving local NF-κB activation, increased aromatase activity, and elevated pro-inflammatory mediators (e.g., TNF-α, IL-1β, IL-6, and PGE2 derived from COX-2) (135). Interestingly, a study indicates that the CD32B-positive adipose tumor margin CLS may be associated with shortened time to metastatic disease (TMD) in patients receiving trastuzumab therapy, potentially serving as a biomarker for personalized treatment strategies in HER2-overexpressing breast cancer (140).

## 5 Mechanisms of adipose tissue inflammation-mediated distant tumor progression

Beyond the two mechanisms highlighted above—cytokine-driven oncogenic signaling and pro-tumorigenic reprogramming of the immune microenvironment—accumulating evidence points to additional systemic routes through which adipose tissue inflammation contributes to tumor initiation and progression, including circulating cytokines and adipokines, free fatty acids and lipid mediators, adipocyte-derived extracellular vesicles (EVs), and gut-derived microbial products (141, 142).

In obesity, enhanced lipolysis together with excessive dietary lipid intake elevates circulating free fatty acids. On one hand, palmitate facilitates tumor dissemination via CD36-mediated fatty acid uptake and protein palmitoylation to maintain oncogenic signaling and metastatic potential (143). On the other hand, it impairs the immunosuppressive capacity of visceral adipose ICOShi Treg cells, further exacerbating systemic metaflammation (120). In addition, obesity-driven lipid dysmetabolism generates various bioactive lipid mediators. For instance, prostaglandin E2 promotes tumor proliferation and invasion (144). These lipid-derived signals can also drive the accumulation of MDSCs and M2-like TAMs via RORγ-dependent transcriptional reprogramming, thereby contributing to a pro-tumor immune microenvironment (145).

Adipocyte-derived EVs serve as an important vehicle for shuttling lipid, protein, and nucleic acid cargo from adipose tissue to distant sites. Their lipid-enriched payload acts as a systemic messenger that connects adipose dysfunction with tumor progression (146). Adipocyte-derived EVs, enriched in glycolytic cargo, reprogram colorectal cancer cell metabolism and promote tumorigenesis (147). Instead, adipocyte EVs in the breast cancer microenvironment sustain mitochondrial oxidative metabolism in cancer cells via HIF-1α (148). Additionally, adipose-derived EVs also carry IL-6 and other inflammatory mediators that enhance tumor cell motility without direct cell contact (149).

Moreover, obesity-associated adipose inflammation converges with gut microbiota dysbiosis and impaired intestinal barrier integrity (150). Obesity-related dysbiosis—characterized by depletion of butyrate-producing taxa, expansion of Gram-negative pathobionts, and disruption of tight junctions and the mucus layer—permits LPS and other microbial metabolites to translocate into the systemic circulation, a state referred to as metabolic endotoxemia (151, 152). Circulating LPS can directly induce PD-L1 expression in tumor cells via Nrf2 signaling and promote an inflamed, immunosuppressive microenvironment, as demonstrated in metabolic syndrome-associated gastric cardia cancer (153). In addition, gut-derived microbial products further modulate systemic immunity and tumor biology via the gut-adipose-tumor axis, a tripartite signaling network that shapes cancer susceptibility and drives tumor progression (153).

Collectively, targeting this pathogenic relay and restoring dysregulated metabolic-inflammatory axes hold great promise for both biomarker discovery and therapeutic intervention against obesity-associated cancers.

## 6 Inflammatory targeted therapy

Anti-inflammatory therapy, such as reducing pro-inflammatory mediators or utilizing endogenous resolution pathways to mitigate inflammation associated with obesity, has emerged as an active area of investigation. Interventions targeting inflammation aim to modulate molecular mechanisms and inflammatory mediators involved in the inflammatory cascade, thereby disrupting the vicious cycle of “inflammation-cancer” and inhibiting tumor progression or enhancing anti-tumor efficacy (154).

The inflammatory TME is characterized by various cytokines and can interact with homologous receptors expressed by the cancer cells themselves, inducing the activation of tumor-promoting signals. The therapeutic targets currently under study include pro-inflammatory cytokines (such as CCL5 and IL-6), cytokine receptors (such as TRAF2), modulation of inflammatory immune cells, blockade of NF-κB signaling pathways, and the CCL2/CCR2 axis (155–158). Several clinical trials targeting IL-6 family cytokines have progressed to Phase I or Phase II, indicating ongoing clinical development (159). Additionally, small molecule inhibitors specifically targeting NLRP3 inflammasomes and other inflammasomes are under research and clinical evaluation (160).

Furthermore, as previously mentioned, immune cells are significant sources of inflammatory cytokines; therefore, immune cells within the TME can serve as therapeutic targets. Certain immune cells produce cytokines that, during chronic inflammation, can act as tumor promoters by influencing cell survival, proliferation, invasion, and angiogenesis. Strategies to block inflammatory pathways and inhibit the infiltration or function of immunosuppressive cells—including MDSCs, Tregs, monocytes, and certain TAM subsets—can restore immune surveillance and promote anti-tumor immunity (72, 161). Additionally, targeting crown-like structures and the molecular marker CD9, which identifies the surface marker of LAMs, highlights a potential therapeutic target for treating obesity-associated metabolic disease (162). Moreover, inducing apoptosis of immunosuppressive TAMs or reprogramming them toward pro-inflammatory, anti-tumor activation states, as well as converting pro-tumor N2-like neutrophils into anti-tumor N1-like neutrophils, represent additional anti-inflammatory, immunomodulatory anti-cancer strategies (163, 164). Nevertheless, such polarization-directed strategies should be applied with caution, because TAM phenotypes span a continuous, context-dependent spectrum rather than discrete M1/M2 states and M1-like TAMs are not universally anti-tumorigenic (165, 166); likewise, the N1/N2 neutrophil dichotomy, while heuristically useful, oversimplifies the functional plasticity of tumor-associated neutrophils (167, 168). Collectively, the obesity-driven, chronically inflamed microenvironment creates a heterogeneous and context-specific immune landscape, calling for plasticity-aware, context-tailored immunomodulatory strategies.

Researchers also highlight the role of diet in mediating inflammation. A Western diet high in saturated fats, processed foods, trans fats, and refined carbohydrates correlates with elevated pro-inflammatory cytokine levels. Conversely, diets rich in fiber and flavonoids, such as anthocyanins, have demonstrated anti-inflammatory effects (169–171). Additionally, natural compounds such as polyphenols—including apigenin, kaempferol, quercetin, curcumin, genistein, isoliquiritigenin, resveratrol, and gallic acid—are capable of modulating several inflammation-related cellular signaling pathways, namely NF-κB, MAPK, Wnt/β-catenin, and PI3K/AKT, through selective intervention on various components of the network. This provides a means for the prevention and treatment of chronic inflammatory diseases (172). Furthermore, certain botanical extracts and traditional Chinese medicine ingredients have been shown to significantly modulate inflammatory responses and inhibit tumorigenesis (173, 174). Despite promising preclinical findings, the clinical translation of these compounds is limited by variable bioavailability, pharmacokinetics, formulation, and dose standardization.

Non-steroidal anti-inflammatory drugs (NSAIDs), including selective COX-2 inhibitors (COXIBs) and nonselective NSAIDs (nsNSAIDs) such as aspirin, celecoxib, ibuprofen, piroxicam, and sulindac, can mediate spontaneous immune-dependent tumor suppression through the COX-2/PGE2 axis (175, 176). The specific mechanism entails COX-2-dependent synthesis of PGE2, which subsequently binds to the PGE2 receptor EP4, initiating intracellular signal transduction pathways. COX-2 plays a role in downregulating the expression of the DNA demethylase TET1, whereas EP4 signaling promotes the upregulation of DNA methyltransferases DNMT1 and/or DNMT3 (177). Changes in the expression of these two epigenetic regulators lead to the silencing of tumor suppressor genes, thereby promoting the occurrence of cancer. NSAIDs or EP4 antagonists may effectively disrupt this inflammation-driven process (175). Additionally, metformin has emerged as a potential anti-inflammatory agent, and anti-cancer therapeutics targeting it are under investigation (178). Although these findings are documented in certain cancers such as colorectal, breast, and lung carcinomas, their applicability to other malignancies remains unclear. Studies investigating the association between NSAID use and cancer risk have yielded mixed results; only half of these studies support the potential cancer risk reduction conferred by NSAIDs, whereas long-term aspirin use does not demonstrate a protective effect against cancer (179). Additionally, a meta-analysis shows that regular aspirin mainly lowers the incidence and mortality of gastrointestinal cancers (notably colorectal and liver cancer), with benefits depending on dose and duration of use, and varying between general populations and high-risk groups (180). Regular long-term use also causes gastrointestinal, renal, cardiovascular, and bleeding adverse events (181). Therefore, future research should focus more on developing novel anti-inflammatory agents with minimized oncogenic side effects.

In recent years, combining anti-inflammatory approaches with conventional chemotherapy or immunotherapy has garnered attention. Immune checkpoint inhibitors and IL-6 pathway blockade have been combined with radiotherapy in clinical trials. For example, ipilimumab (an anti-CTLA-4 antibody) and nivolumab (an anti-PD-1 antibody), together with tocilizumab (an IL-6 receptor-blocking antibody), were administered with stereotactic body radiotherapy in patients with refractory pancreatic cancer, with the combination designed to alter the TME and circumvent immune evasion mechanisms (182). Furthermore, monoclonal antibodies (mAbs) targeting cytokines or their receptors, as well as small molecule inhibitors or antagonists that disrupt ligand-receptor signaling complexes or downstream JAK-STAT pathways, have been demonstrated to enhance outcomes in immune checkpoint blockade (ICB) therapy (183).

A novel nanotechnology approach based on developing standardized chemotherapeutic nanoparticle delivery systems holds considerable promise as an anti-inflammatory strategy. Several nanoparticle drug delivery platforms have been designed, including Hesperidin-loaded gold nanoparticles (Hsp-AuNPs), nanoparticles composed of clove bud extract and zinc oxide (CBE-ZnO-NPs), ruthenium oxide nanoparticles (RuO2 NPs), T-lipos-CPAuNCs, and MX@Arg-Gd-MPDA (MAGM NPs). They possess significant potential for oncologic therapy through photothermal tumor ablation, suppression of pro-inflammatory cytokine secretion, targeted delivery of anti-inflammatory agents, and controlled release mechanisms (184–188). **Figure 3** summarizes the application of these anti-inflammatory therapies. **Table 2** provides a summary of inflammation-targeted therapeutic strategies for cancer management among obese patients.

**Figure 3:**
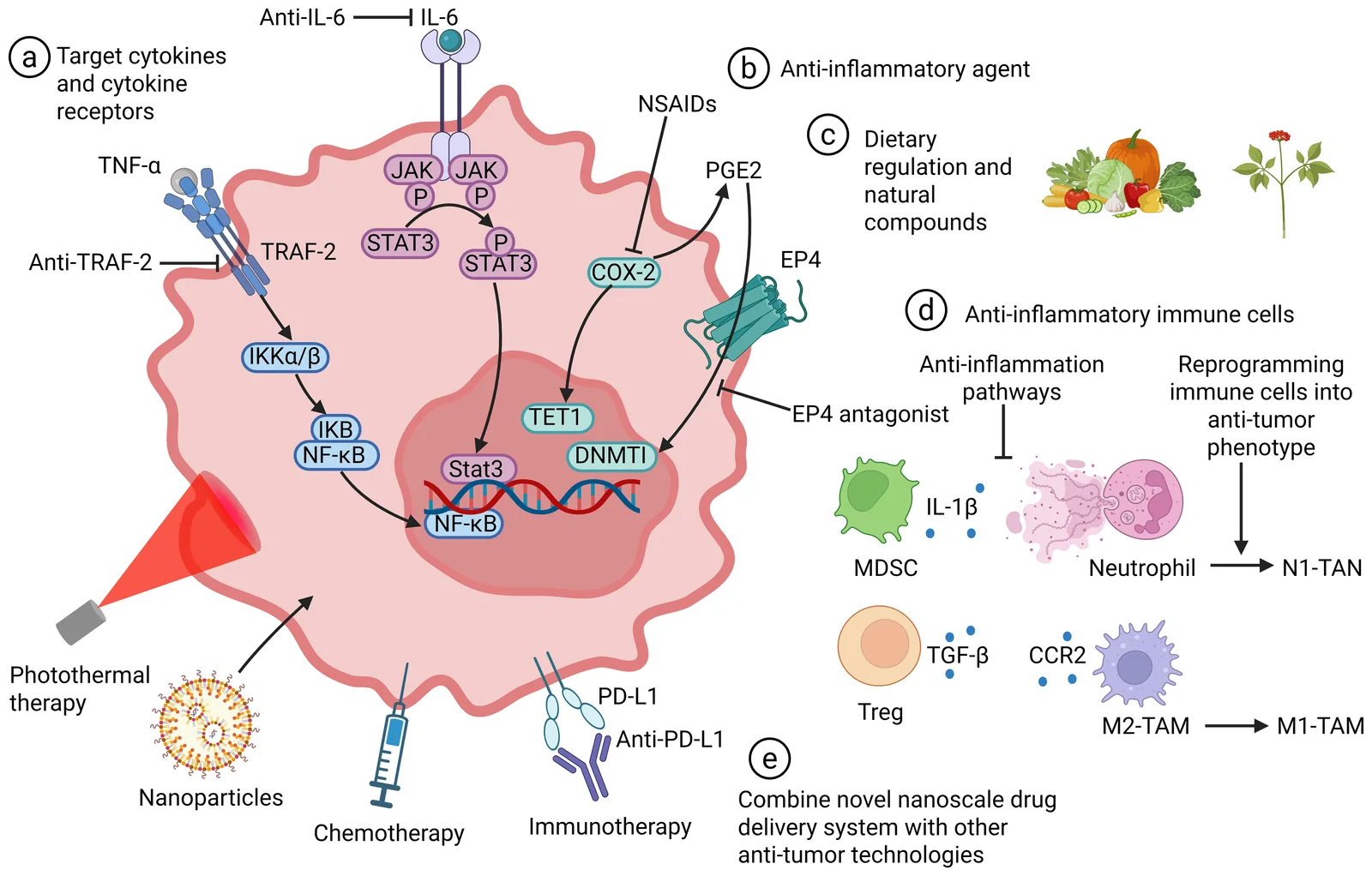
The anti-inflammation therapy for cancer treatment in obese patients. **(a)** Target cytokines (e.g., IL-6) and cytokine receptors (e.g., TRAF-2); **(b)** Anti-inflammatory agent (e.g., NSAID) and EP4 antagonist; **(c)** Incorporate natural anti-inflammatory compounds into the diet and utilize plant extracts; **(d)** Suppress immune cells from producing inflammatory cytokines or reprogram immune cells into anti-inflammatory and anti-tumor phenotypes; **(e)** Combine novel nanoscale drug delivery systems with other technologies (e.g., photothermal therapy, chemotherapy, and immunotherapy). TRAF-2, TNF receptor-associated factor 2; NSAID, non-steroidal anti-inflammatory drug; COX-2, Cyclooxygenase-2; MDSC, myeloid-derived suppressor cell; PD-1, programmed cell death 1; PD-L1, programmed cell death ligand 1; CCR2, C-C motif chemokine receptor 2.

**Table 2:** Summary of inflammation-targeted therapies for cancer treatment in obese patients.

Therapeutic category	Agent/strategy	Molecular target/mechanism	Therapeutic benefits	References
Cytokine & Receptor Blockade	Tocilizumab & IL-6 inhibitors	Tocilizumab (anti-IL-6 receptor mAb) and related agents block IL-6/IL-6R signaling; combined with immune checkpoint inhibitors (ipilimumab [anti-CTLA-4], nivolumab [anti-PD-1]) in combination regimens	Prevents oncogenic JAK-STAT activation; reverses tumor immune evasion during radiotherapy	(159, 182, 183)
	Liquidambaric acid	Targets TRAF2 (TNF receptor-associated factor 2)	Inhibits Wnt/β-catenin and NF-κB signaling; suppresses tumor growth in colon cancer models	(156)
	CCR2 antagonists/CCL2 inhibitors	Blocks CCL2/CCR2 axis on infiltrating monocytes	Prevents recruitment of pro-inflammatory monocytes and myeloid-derived suppressor cells; restores immune surveillance	(157)
Inflammasome Inhibition	NT-0249, NT-0796 & NLRP3 inhibitors	Inhibits NLRP3 complex assembly and activation	Reverses high-fat diet-induced systemic inflammation; reduces IL-1β cleavage; suppresses tumor-promoting angiogenesis	(93, 94)
NSAIDs & Metabolic Agents	Aspirin, Celecoxib, Ibuprofen, Sulindac	Inhibits COX-2–derived PGE2 synthesis	Disrupts COX-2/PGE2/EP4 pathway; restores TET1 and inhibits DNMT1/3 to prevent epigenetic silencing of tumor suppressors	(175–177)
	Metformin	Activates AMPK cascade	Alleviates obesity-induced metabolic stress; suppresses EMT and metastatic dissemination	(178, 179)
Dietary Phytochemicals	Polyphenols (Curcumin, Resveratrol, Quercetin)	Multi-target modulation of NF-κB, MAPK, Wnt, and PI3K/AKT pathways	Dietary chemopreventive agents; reduce circulating CRP, TNF-α, and IL-6; resolve chronic tissue inflammation	(170–172)
	Berberine	Alleviates chronic mucosal and systemic low-grade inflammation	Repurposed anti-inflammatory TCM agent; prevents colitis-associated tumorigenesis	(174)
Immune Cell Reprogramming	TAM & Neutrophil Reprogramming	Drives immunosuppressive TAMs toward pro-inflammatory, anti-tumor activation states and N2-like neutrophils toward N1-like anti-tumor phenotypes	Reprograms TAMs and neutrophils into anti-tumor immunogenic phenotypes; restores immune surveillance	(163, 164)
Nanotechnology Platforms	Hsp-AuNPs (Hesperidin-loaded gold NPs)	Standardized chemotherapeutic nanoparticle delivery	Induces biocompatible phagocytosis; suppresses systemic pro-inflammatory cytokines; mediates tumor cell ablation	(184)
	CBE-ZnO-NPs (Clove bud extract–ZnO NPs)	Multifunctional green-synthesized drug delivery platform	Targeted delivery of botanical anti-inflammatory agents; potent antibacterial and antineoplastic activity	(185)
	MAGM NPs (Mesoporous polydopamine)	MRI-guided photothermal therapy with controlled drug release	Delivers anti-inflammatory drugs; enables image-guided localized photothermal tumor ablation; minimizes systemic toxicity	(187)
	RuO_2_ NPs & T-lipos-CPAuNCs	ML-optimized nanomedicines	Reduces off-target inflammation; overcomes therapeutic resistance; provides controlled, targeted tumor suppression	(186, 188)

## 7 Conclusions and discussions

Our literature review found that different studies employ varying criteria for defining obesity. Certain individuals classified as overweight or obese retain normative metabolic profiles, exhibiting a reduced risk for cardiovascular disease (CVD) and type 2 diabetes, referred to as MHO. This contrasts with metabolically MUO individuals or those with metabolic syndrome (189). Furthermore, the incidence of severe obesity (defined as BMI ≥ 40 kg/m²) exceeds that of moderate obesity. Relying solely on BMI as an obesity metric may be insufficient, as an elevated waist circumference can persist even when BMI or total body fat is normalized, still presenting features such as insulin resistance, impaired glucose tolerance, and heightened inflammatory responses (190). The etiologies of obesity are multifaceted, closely linked to adipose tissue distribution—including VAT and SAT—and adipose tissue types—WAT versus BAT—which are associated with distinct metabolic consequences. Under pathological conditions, VAT and WAT are more prone to promote metabolic diseases leading to mortality, whereas SAT and BAT exert protective effects against components of metabolic syndrome. These complexities have profoundly impacted the definition and treatment of obese patients (191). Recently, more precise anthropometric and bioelectrical impedance measurements—such as waist circumference, waist-to-hip ratio, and body fat percentage—have been developed. Therefore, complementary measures should be combined for a comprehensive assessment (192). This underscores the necessity of utilizing multiple phenotypic indicators in clinical practice for accurate obesity characterization and decision-making.

Recent years have brought considerable advances in unraveling the molecular basis of obesity-associated inflammation and translating these findings into clinical biomarkers. Mechanistically, obese adipose tissue harbors spatially organized, self-reinforcing inflammatory niches, wherein adipocytes undergo fibro-inflammatory transcriptional reprogramming (193). Obesity also confers long-lasting epigenetic and metabolic imprints, termed maladaptive trained immunity, on adipose tissue macrophages and their progenitors. This sustains pro-inflammatory responses even after weight loss, offering a molecular rationale for the “metabolic memory” associated with obesity (194, 195). Epitranscriptomic control via N6-methyladenosine (m^6^A) modification of inflammatory transcripts, innate immune sensing of the Nrf2-cGAS-STING axis, and microRNA-mediated intercellular communication represent underappreciated amplifiers of adipose inflammation (196–198). Collectively, these findings highlight that obesity-driven metaflammation originates from an integrated network of metabolic, epigenetic, and innate-immune signals, not a single cytokine cascade. Meanwhile, biomarker development has extended beyond classical markers (CRP, IL-6, TNF-α) to include adipokine panels, circulating microRNAs, and composite inflammatory scores (199, 200). Importantly, prospective studies link inflammatory biomarkers to disease risk in obesity. Systemic inflammation partially mediates the obesity-colorectal cancer association (201); YKL-40 (an inflammatory macrophage activation marker) independently predicts obesity-related cancer incidence in patients with early type 2 diabetes (202); and severe obesity, together with high CRP and insulin resistance, yields maximal all-cause mortality and site-specific cancer risk (203). These findings support the promise of inflammation-based risk stratification in obesity, though most candidate biomarkers still await prospective validation.

Obese patients should first be advised to undergo weight loss treatment to reduce cancer risk; for patients with obesity-related tumors, weight loss treatment is beneficial for improving the long-term efficacy of immunotherapy. Traditional weight loss treatments include dietary interventions (such as the ketogenic diet), exercise, drug therapy (e.g., the GLP-1 receptor agonist semaglutide, the lipase inhibitor orlistat, and metformin), behavioral therapy, and bariatric surgery (191, 204–206). Interestingly, some of these weight loss therapies can also exert anti-tumor effects, such as dietary changes, lifestyle interventions, and GLP-1 receptor agonists (207–209). This may provide new ideas and directions for future tumor treatment, as early weight management in obese patients can intervene in tumor progression in such patients. Recent studies have also shown that probiotic supplementation can modulate gut microbiota dysbiosis in obese patients, conferring potential metabolic and anti-inflammatory benefits (210, 211). Importantly, a new concept—precision obesity medicine—emphasizes personalized, whole-life-cycle clinical decision-making for obese patients (212). In clinical practice, obesity status should be taken into account when managing patients with cancer, and guideline-based treatment strategies should be provided so that this population can achieve the dual benefits of weight control and cancer treatment as far as possible.

This review outlines a mechanistic pathway linking obesity to tumorigenesis: obesity induces systemic chronic inflammation, characterized by elevated levels of inflammatory cytokines and activation of associated signaling pathways, triggering downstream cascades. These processes exacerbate inflammation-driven oxidative stress, cellular proliferation, angiogenesis, and activation of oncogenic signaling. Concurrently, pro-inflammatory activation states of immune cells foster a tumor-promoting inflammatory microenvironment, facilitating immune evasion by tumor cells. In this environment, cytokines secreted by adipose tissue and small molecules stimulated by inflammatory immune cells engage in extensive crosstalk with tumor cells, contributing to tumor initiation and progression. Subsequently, building upon existing research and recent scientific trends, we summarize inflammation-targeted therapies for cancer. These include combinations of anti-inflammatory cytokine inhibitors with chemotherapy and immunotherapy; targeting pro-inflammatory receptors and signaling pathways; reprogramming inflammatory immune cells; utilizing natural anti-inflammatory compounds; and the nanodelivery of anti-inflammatory drugs. Most of these strategies remain at the animal-experimental or early-phase clinical stage. Although some have shown encouraging signals in preclinical models or early-phase trials, their efficacy and safety in obese patients with cancer require rigorous validation. Moving forward, continuous exploration of novel therapeutic modalities is essential to address systemic inflammation and tumor risk in obese patients, and may ultimately inform safe and effective, personalized intervention strategies.

Despite strong preclinical rationale for targeting obesity-associated inflammation, anti-inflammatory strategies warrant careful consideration. Global suppression of systemic inflammation entails safety trade-offs. In the CANTOS trial, IL-1β inhibition by canakinumab lowered lung cancer incidence yet increased fatal infections, highlighting the balance between blocking pathological inflammation and maintaining host defense (213). Similarly, cytokine-targeted agents such as anti-TNF-α and anti-IL-6R antibodies may blunt antitumor immunity and impair tissue-repair capacity (214). Adding further complexity, IL-1, IL-6, TNF-α, and NLRP3 inflammasome signaling are not universally pro-tumorigenic, and their net effects are highly context- and tumor-type-dependent. For instance, TNF-α may drive either tumor clearance or progression; inflammasome-driven pyroptosis exerts dual roles that can boost antitumor immunity or sustain malignant progression; and NLRP3 signaling can promote or restrain gastrointestinal cancers, with consequences for checkpoint-inhibitor responses (215–217). Collectively, indiscriminate systemic anti-inflammatory approaches are not recommended. Therapeutic strategies should be tailored to tumor type, biological context, and disease stage to selectively restrain pathological inflammation without abrogating protective immunity and tissue repair (218, 219).
